# Identifying additional studies for a systematic review of retention strategies in randomised controlled trials: making contact with trials units and trial methodologists

**DOI:** 10.1186/s13643-017-0549-9

**Published:** 2017-08-22

**Authors:** Valerie Brueton, Jayne F. Tierney, Sally Stenning, Greta Rait

**Affiliations:** 1 Florence Nightingale Faculty of Nursing and Midwifery, Department of Adult Nursing, King’s College London, 57 Waterloo Road, London, SE1 8WA United Kingdom; 2 MRC Clinical Trials Unit at UCL, Aviation House, 125 Kingsway, London, WC2B 6NH United Kingdom; 3 PRIMENT Clinical Trials Unit, Research Department of Primary Care and Population Health, Royal Free and University College Medical School, Rowland Hill Street, London, NW3 2PF United Kingdom

**Keywords:** Systematic review methods, Unpublished data, Searching for methodological research, Survey, Personal communication, Methodology review

## Abstract

**Background:**

Search strategies for systematic reviews aim to identify all evidence relevant to the research question posed. Reports of methodological research can be difficult to find leading to biased results in systematic reviews of research methodology. Evidence suggests that contact with investigators can help to identify unpublished research. To identify additional eligible randomised controlled trials (RCTs) for a Cochrane systematic review of strategies to improve retention in RCTs, we conducted a survey of UK clinical trials units (CTUs) and made contact with RCT methodologists.

**Methods:**

Key contacts for all UK CTUs were sent a personalised email with a short questionnaire and summary protocol of the Cochrane methodology review. The questionnaire asked whether a RCT evaluating strategies to improve retention embedded in a RCT had ever been conducted by the CTU. Questions about the stage of completion and publication of such RCTs were included. The summary protocol outlined the aims, eligibility criteria, examples of types of retention strategies, and the primary outcome for the systematic review. Personal communication with RCT methodologists and presentations of preliminary results of the review at conferences were also used to identify additional eligible RCTs. We checked the results of our standard searches to see if eligible studies identified through these additional methods were also found using our standard searches.

**Results:**

We identified 14 of the 38 RCTs included in the Cochrane methodology review by contacting trials units and methodologists. Eleven of the 14 RCTs identified by these methods were either published in grey literature, in press or unpublished. Three remaining RCTs were fully published at the time. Six of the RCTs identified were not found through any other searches. The RCTs identified represented data for 6 of 14 RCTs of incentive strategies (52% of randomised participants included in the review), and 6 of 14 RCTs of communication strategies (52% of randomised participants included in the Cochrane review). Data were unavailable for two of the RCTs identified.

**Conclusions:**

Methodological evaluations embedded in RCTs may be unpublished, published in the grey literature or where published, poorly indexed in bibliographic databases. To identify such studies and minimise selection bias in systematic reviews of methodological evaluations, reviewers should consider contacting CTUs and trial methodologists.

**Electronic supplementary material:**

The online version of this article (doi:10.1186/s13643-017-0549-9) contains supplementary material, which is available to authorized users.

## Background

Search strategies for systematic reviews are designed to find all of the relevant evidence to answer a specific research question [1] and to minimise bias [2]. However, capturing all eligible studies for a systematic review through bibliographic database searching alone can be difficult. This is because studies might be poorly indexed, or the search strategy may not include all the necessary terms, or instead because the studies are hidden in grey literature. Moreover, methodological studies embedded within trials (SWATs) or other studies may be even less accessible. Unpublished studies, in particular, can be difficult to identify, and failure to include these and other difficult to find studies in systematic reviews can lead to under or overestimation of effects [3]. While there is evidence to suggest that contacting investigators is a useful way to find published and unpublished eligible studies that are otherwise difficult to find for systematic reviews of interventions [1, 2], we have not found any examination of different ways of identifying unpublished eligible studies.

To identify additional studies for a Cochrane methodology systematic review of strategies to improve retention in randomised controlled trials RCTs [4], we conducted a survey of UK clinical trial units (CTUs) and communicated with colleagues working in RCT methodology, as well as undertaking standard searches of bibliographic databases, conference abstracts and reference lists. In this paper, we describe the methods and the results of using these additional search methods.

## Methods

For the survey, we used the UK Clinical Research Collaboration website (UKCRC), http://www.ukcrc-ctu.org.uk/ [5] to identify a key contact for each UK CTU. The contact details for each key contact were entered into a Microsoft Excel spreadsheet. We sent a personalised email to each CTU key contact with a two-page summary protocol of the Cochrane review attached. The summary protocol outlined the purpose of the review, the inclusion criteria for eligible RCTs, examples of different types of retention strategies used in RCTs, and a definition of the primary outcome for the review, i.e. retention of participants in RCTs. We also sent a short questionnaire (Additional file 1: Appendix 1) asking whether a RCT of strategies to improve retention embedded within a RCT had ever been conducted by the CTU. For CTUs that had conducted such RCT/s, further questions were asked about the stage of completion of the RCT, availability of the RCT protocol, and the publication status. A reminder email was sent to CTU contacts who had not responded after 4 weeks. The survey was conducted in April 2010 between the initial bibliographic database searches for the review which were conducted in February 2009 and before the bibliographic database search updates were conducted in May 2012.

In addition to conducting the survey, we discussed the review with colleagues and contacts we knew in the field of RCT methodology. We also presented a poster of the preliminary results of the review at the Society for Clinical Trials 31st International conference [6]. The poster mentioned that we wanted to identify additional RCTs for the review. We also posted a message on the conference notice board directing delegates to visit our poster and asking for information about any known potentially eligible RCTs (Additional file 2: Appendix 2). Preliminary results were also presented at the 1st UK Medical Research Council (MRC) Methodology conference [7]. At both conferences, questionnaires similar in format to those sent to UK CTUs were available for conference delegates to complete.

We checked to see if eligible studies identified through these additional methods were also found through our standard searches and if any had been missed.

### Data management

We recorded responses to the survey in Microsoft Excel. For potentially eligible RCTs identified, details of the publication status, and if published, details of the author, year of publication, title and journal were recorded. For unpublished potentially eligible RCTs in progress or completed, details of the RCT title and principal investigator were recorded. We recorded the same details for all potentially eligible RCTs identified through personal communication.

Full copies of each published potentially eligible RCT were sourced, screened for eligibility using methods described previously [4] and subsequently sent to a second reviewer (GR). For each eligible retention RCT, we proceeded with the data extraction also described previously [4]. We emailed the principal investigator linked with any eligible and unpublished RCT to see if they were willing to share data to include in the review.

## Results

### Survey results

Sixty-nine per cent (34/49) of UK CTUs responded to the survey; 22 (45%) of which responded to the initial email, and 12 (24%) CTUs to the reminder email. Sixteen potentially eligible studies were identified. These studies were at different stages of progression, i.e. planned, in progress, completed and unpublished, or completed and published. Seven of these RCTs were eligible for inclusion in the systematic review of retention strategies. At the time of the survey, two RCTs were fully published [8, 9], one was in press [10], two were published in grey literature: one as a PhD thesis chapter [11], and one as an appendix to a Health Technology Assessment (HTA) review [12] (Table 1). The results of two RCTs were unpublished at the time (Table 1). Results for one of these RCTs have since been published [13]. Data for one unpublished RCT and one published RCT identified through the survey were not available for the Cochrane systematic review analysis (Table 2). Investigators provided data for five RCTs allowing these RCTs to contribute to the meta-analyses of incentives and communication strategies (Table 2). Investigators also provided information that contributed to a risk of bias assessment on those five RCTs [4].

**Table 1:** Additional RCTs identified through contact with CTUs and trial methodologists for a systematic review of strategies to improve retention in RCTs

Eligible RCT	Publication status at the time of the survey	Journal	Duplicate sources
Survey of UK CTUs			
Cockayne (2005) [9]	Published	BMC Medical Research Methodology	Reference list of relevant literature [19]
Brown (1997) [8]	Published	Journal of Epidemiology and Community Health	Reference list of relevant literature and included RCTs [19, 20]
Marson (2007) [12]	Grey literature	Health Technology Assessment (HTA) report Appendix	–
Nakash 2007 [11]	Grey literature	PhD thesis chapter	–
Ashby 2011 [10]	In press	Journal of Clinical Epidemiology	Medline search update (May 2012)
MacLennan [13]	Unpublished	Subsequently published in BMC Trials 2014	–
Mitchell	Unpublished on-going study	–	–
Personal communication			
Gates (2009) [14]	Published	Trials	Medline search updates (May 2012) Cochrane Methodology register
Khadajesari (2011) (a) [15]	Unpublished at the time of the survey	Subsequently published in Journal of Medical Internet Research	Medline search updates (May 2012)
Khadajesari (2011) (b) [15]	Unpublished at the time of the survey	Subsequently published in Journal of Medical Internet Research	Medline search updates (May 2012)
Severi (2011) (a) [16]	Unpublished at the time of the survey	Subsequently published in Clinical Trials	Medline search updates (May 2012) Survey of UK CTUs
Severi (b) (2011) [16]	Unpublished at the time of the survey	Subsequently published in Clinical Trials	Medline search updates (May 2012) Survey of UK CTUs
Bailey 1	Unpublished	–	–
Bailey 2	Unpublished

**Table 2:** Data provided by additional RCTs identified through contact with CTUs and trial methodologists for a systematic review of strategies to improve retention in randomised trials

Eligible RCT	RCT comparison	Number of participants results are based on
Meta-analysis of incentive strategies		
Cockayne (2005) [9]	Addition of offer of non-monetary incentive versus no offer	1038
Brown (1997) [8]	Entry into a prize draw versus no entry into a prize draw	No data available
Gates (2009) [14]	Monetary incentive versus no incentive	2144
Khadajesari (2011) (a) [15]	Addition of offer of monetary incentive/ entry into prize draw/monetary donation to charity with reminder email versus no offer with reminder email	1837
Khadajesari (2011) (b) [15]	Addition of offer of monetary incentive with reminder email versus no offer with reminder email	2591
Bailey 1	Addition of £20 voucher versus addition of £10 voucher	417
Bailey 2	Addition of £10 voucher plus offer of £10 voucher versus addition of £5 plus offer of £5	485
Total		8521
Meta-analysis of communication strategies		
Marson (2007) [12]	Enhanced letter versus standard letter	1815
Nakash 2007 [11]	Additional reminder versus usual follow-up procedures	298
Ashby 2011 [10]	Additional reminder versus usual follow-up procedures	148
MacLennan [13]	Additional reminder versus usual follow-up procedures	753
Mitchell	Pre contact and participant up-date via newsletters	No data available
Severi (2011) (a) [16]	Text message reminder plus a fridge magnet versus standard follow-up	1950
Severi (b) (2011) [16]	Reminder telephone call from principal investigator versus standard follow-up	127
Total		5091

Duplicate sources of the RCTs identified by the survey are shown in Table 1. One RCT in press at the time of the survey was subsequently published [10] and found in our Medline search updates. Two eligible published RCTs [8, 9] were found in the reference lists of RCTs included in the review [8] and relevant literature [8, 9]. The two RCTs published in the grey literature [11, 12] and both unpublished RCTs (by Mitchell and Maclennan) were not identified through any other source.

### RCTs identified through personal communication

Seven eligible RCTs (from three publications) were identified in the first instance through personal communication [14–16] (two Bailey unpublished reports). One RCT was identified through contact with colleagues in the Hubs for Trials Methodology Research [14], https://www.methodologyhubs.mrc.ac.uk/about/hubs/ [17]. The other six RCTs were identified through contact with colleagues at CTUs locally either before the survey was conducted [15] or afterward (Bailey, unpublished, [16]). The contribution of these trials to the meta-analyses in the systematic review is shown in Table 2. Two of the RCT publications identified through personal communication reported two RCTs each [15, 16]. All of the published eligible RCTs identified through personal communication were found in our Medline search updates [14–16]. One of the RCTs was also identified by the survey of CTUs [16], and another published RCT was identified through the Cochrane methodology register [14]. The two unpublished RCTs by Bailey were not identified through any other source.

The additional RCTs identified by the survey and through personal communication with methodologists represented data for 6 of 14 RCTs of incentive strategies (52% of the randomised participants for incentive strategies included in the Cochrane review) and 6 of 14 RCTs of communication strategies (52% of randomised participants for communication strategies included in the review) (Table 2) [4].

## Discussion

The survey of CTUs and personal communication with colleagues in the field of RCT methodology helped us identify 14 of the 38 RCTs included in the Cochrane systematic review of strategies to improve retention in RCTs [4]. Eleven of the 14 RCTs identified were either published in grey literature, in press, or unpublished at the time. Three of the RCTs identified were published. Six of the 14 RCTs identified were not identified through any other source. We found that principal investigators were willing to contribute results from unpublished RCTs, which made a major contribution to the meta-analyses of incentive and communication strategies.

### Strengths

To our knowledge, this is the first time a survey of CTUs and personal communication with trial methodologists were combined to identify eligible studies to include in a Cochrane methodology review. The response rate to the survey was high(69%) and may be explained in part by the importance given to retention in RCTs by UK CTUs and by using an individualised approach to the survey with a short questionnaire. We also raised the profile of the review by presenting preliminary results and giving details of the survey at trials methodology conferences, which may explain the principal investigators willingness to share unpublished data. As the survey was conducted between the initial database search and the search updates, we were able to check the reliability of our other search strategies in picking up these RCTs and see a net gain of using these extra search methods. The inclusion of additional RCTs improved the overall quality of the review results.

### Limitations

Only one reminder email was sent to the CTUs surveyed. More reminders may have improved the number of responses. It is therefore possible that more unreported retention RCTs could have been identified at the time were more reminders sent. As the survey was conducted in the UK, the results do not include RCTs in progress or unpublished from other regions. Although presentations of the preliminary results of the review at national and international conferences helped to publicise and draw attention to the review inclusion criteria, no new eligible retention RCTs were identified through these means. We checked the results of our initial database searches and found that two of the eligible RCTs identified through the survey were not identified through the initial database searches [8, 9]. This may be explained by a mismatch between the search terms used for our initial database searches, ambiguity around definitions of retention and attrition in RCTs, and indexing terms used to label subjects by bibliographic databases when studies were indexed.

### Implications of the results

Personal communication has been used before in methodology reviews to identify unpublished data [2], and the evidence suggests that email is the best method to use [2]. The UKCRC [5] website was a useful resource for identifying key contact details for each CTU. We found that conducting the survey by email was efficient; nevertheless, systematic reviewers planning to include such a survey as part of their search strategy may wish to consider the associated additional time and costs. Since our survey was conducted, the Studies Within a Trial (SWAT) database of evaluations of methods for RCTs has been established [18]. Systematic reviewers could also consider searching this resource to identify embedded methodology RCTs in progress for future systematic reviews of research methodology.

It is clear from the results of the survey that RCTs evaluating strategies to improve retention in RCTs remain unpublished or are published in the grey literature. A possible explanation for this is the priority given to publishing the results of host RCTs. Moreover, as these RCTs of retention strategies are embedded within RCTs of health interventions, they have less prominence within a trials report and are likely to be more difficult to publish as stand-alone pieces of research. Thus, clear guidance on the reporting of methodology RCTs embedded in RCTs is needed, which in turn might ensure more accurate indexing in bibliographic databases.

Published eligible RCTs identified through the survey and by personal communication were mostly published since 2005 in journals focused on research methodology, e.g. BMC Medical Research Methodology [9], Clinical Trials [16], Trials [14], BMC Trials [13], Journal of Medical Internet Research [15], and Journal of Clinical Epidemiology [10] (Table 1). This reflects the relatively recent emergence of trial and other research methodology as distinct areas for research. Therefore, hand searching of relevant research methodology journals that are not included in major bibliographic databases would be useful for methodology reviews such as ours. The other RCTs identified for the systematic review were published in journals that reflected the focus of the host trial within which the retention trial was embedded. Some were published in general medical journals, e.g. the British Medical Journal, or in more specific clinical journals such as, Clinical Oncology, Journal of Health Psychology, Child Maltreatment and Gerontologist, reflecting the spectrum of host trials that the retention trials were embedded within. Thus, search strategies to identify SWATs for methodology reviews will need to remain broad and continue to make use of the major medical bibliographic databases.

## Conclusion

Methodological evaluations, particularly those embedded in RCTs or other clinical studies, may be unpublished, published in the grey literature or where published, poorly indexed in bibliographic databases. To identify such studies and minimise selection bias in systematic reviews of methodological evaluations, reviewers should consider contacting CTUs and trial methodologists.

## Additional files

Additional file 1: Appendix 1. UK CTU Survey Questionnaire. (DOCX 15 kb)

Additional file 2: Appendix 2. Message posted on the Society for Clinical Trials 31st Meeting (2010) conference message board. (DOCX 14 kb)

## Abbreviations

BMC: BioMed Central
CTU: Clinical Trials Unit
HTA: Health Technology Assessment
MRC: Medical Research Council
RCT: Randomised Controlled Trial
UK: United Kingdom
UKCRC: United Kingdom Clinical Research Collaboration
