# Supplementary material for: Identifying additional studies for a systematic review of retention strategies in randomised controlled trials: making contact with trials units and trial methodologists
Source: Syst Rev. 2017 Aug 22;6:167. doi: 10.1186/s13643-017-0549-9 (PMC5568351; doi:10.1186/s13643-017-0549-9)
Supplement: Supplementary file 1 — Appendix 1. UK CTU Survey Questionnaire. (DOCX 15 kb) [file 13643_2017_549_MOESM1_ESM.docx]

**Additional file 1: Appendix 1: UK CTU Survey Questionnaire**

**Contact person:**

**Name of CTU:** _____________________________________________________

1. **Have you ever conducted a randomised trial of strategies to reduce attrition?**

(For example this could be a trial comparing incentives with usual follow-up procedures or a trial comparing two different types of follow-up strategy)

Yes  If **Yes** go to question 2

No  If **No** please return this form by fax or email **(see details below)**

1. **Is this trial a randomised trial embedded within another randomized trial?**

(For example this could be a trial comparing incentives with usual follow-up procedures embedded within a randomized trial comparing two treatments for hypertension)

Yes  If **Yes** go to question 3

No  If **No** please return this form by fax or email **(see details below)**

**3**. **Is this trial completed?**

Yes  If **Yes** go to question 4

No  If **No** go to question 5

**4. Is there an up to date reference for this trial?**

Yes  If **Yes** please supply an up to date reference for the trial.

Enter the up to date reference for your trial here:

No  If **No** go to the question 5

**5**. **Can you supply a trial protocol?**

Yes  If **Yes** please supply a copy of the trial protocol.

Enter the name of the trial protocol here:

No  If **No** please return this form by fax or email

________________________________________________

Please return this form by fax or email
