# Supplementary material for: Identifying additional studies for a systematic review of retention strategies in randomised controlled trials: making contact with trials units and trial methodologists
Source: Syst Rev. 2017 Aug 22;6:167. doi: 10.1186/s13643-017-0549-9 (PMC5568351; doi:10.1186/s13643-017-0549-9)
Supplement: Supplementary file 2 — Appendix 2. Message posted on the Society for Clinical Trials 31st Meeting (2010) conference message board. (DOCX 14 kb) [file 13643_2017_549_MOESM2_ESM.docx]

**Additional file 2: Appendix 2 Message posted on the Society for Clinical Trials 31st Meeting (2010) conference message board**

**Poster number P87: Strategies to reduce attrition from randomised trials**

Do you know of any RCTs eligible for this systematic review?

We are looking for more RCTs within which are embedded RCTs evaluating strategies to reduce attrition.

Completed, published or unpublished (but let us know if you have an ongoing trial)

Randomised or quasi randomised

Comparing one or more strategies to reduce attrition or comparing one or more strategies with no strategy

If you have any RCTs, please contact us.

**Or come to Poster P87 and pick up a leaflet and complete a form**
